# Sentence comprehension in patients with dementia of the Alzheimer’s type

**DOI:** 10.7717/peerj.8181

**Published:** 2019-12-03

**Authors:** Xinmiao Liu, Wenbin Wang, Haiyan Wang, Yu Sun

**Affiliations:** 1 School of English for Specific Purposes, Beijing Foreign Studies University, Beijing, China; 2 National Research Centre for Foreign Language Education, Beijing Foreign Studies University, Beijing, China; 3 Institute of Language and Brain Science, School of Translation Studies, Qufu Normal University, Rizhao, China; 4 Department of Neurology, Xuanwu Hospital of Capital Medical University, Beijing, China

**Keywords:** Alzheimer’s disease, Language deficit, Chinese, Language comprehension

## Abstract

Sentence comprehension is diminished in patients with dementia of the Alzheimer’s type (DAT). However, the underlying reason for such deficits is still not entirely clear. The Syntactic Deficit Hypothesis attributes sentence comprehension deficits in DAT patients to the impairment in syntactic ability, whereas the Processing Resource Deficit Hypothesis proposes that sentence comprehension deficits are the result of working memory deficiency. This study investigated the deficits in sentence comprehension in Chinese-speaking DAT patients with different degrees of severity using sentence-picture matching tasks. The study revealed a significant effect of syntactic complexity in patients and healthy controls, but the effect was stronger in patients than in healthy controls. When working memory demand was minimized, the effect of syntactic complexity was only significant in patients with moderate DAT, but not in healthy controls or those with mild DAT. The findings suggest that in patients with mild DAT, working memory decline was the major source of sentence comprehension difficulty and in patients with moderate DAT, working memory decline and syntactic impairment jointly contributed to the impairments in sentence comprehension. The source of sentence comprehension deficits varied with degree of dementia severity.

## Introduction

Sentence comprehension has been found to diminish in dementia of the Alzheimer’s type (DAT) patients in various tasks, such as sentence-picture matching (Rochon, Waters & Caplan, 1994), enactment (Emery, 1983, 1988), or the Token Test (Tomoeda et al., 1990). Sentence comprehension is impaired even at the very early stage of the disease (Bates, Wulfeck & MacWhinney, 1991; Grober & Bang, 1995; Kempler et al., 1998; Kontiola et al., 1990; Leikin & Aharon Peretz, 1998; MacDonald et al., 1996; Rochon, Waters & Caplan, 1994; Small, Kemper & Lyons, 1997; Tomoeda et al., 1990). In spite of all these efforts to identify the pattern of comprehension deficits in DAT patients, the exact cause of the impairment is still far from clear (Miller, 1989; Flanagan et al., 2017; Venneri et al., 2018). Sentence comprehension is a complex process involving both linguistic processes such as syntactic analysis and extra-linguistic processes including working memory. The impairment in either of these processes may lead to sentence comprehension deficits (Kempler et al., 1998; Small, Andersen & Kempler, 1997; Montgomery, Gillam & Evans, 2016). Thus, it is still not clear whether sentence comprehension deficits are caused by working memory dysfunction or syntactic decline.

So far there have been two divided views regarding the reasons for sentence comprehension impairments in DAT patients, namely the Syntactic Deficit Hypothesis (Emery, 1985; Grober & Bang, 1995) and the Processing Resource Deficit Hypothesis (Rochon, Waters & Caplan, 1994; Waters, Caplan & Hildebrandt, 1991; Waters, Caplan & Rochon, 1995). The Syntactic Deficit Hypothesis attributed sentence comprehension deficits to the impairment in syntactic ability. In this theory, *syntactic deficit* or *syntactic decline* refers to the difficulty in accessing or using syntactic knowledge, typically reflected by a decline in syntactic abilities such as syntactic analysis or computation. Syntactic deficit is a type of competence decline which underlies many specific performance deficits such as sentence production deficits, sentence comprehension deficits or even discourse comprehension deficits. Grober & Bang (1995) examined the effect of semantic constraints, syntactic complexity and working memory demand on sentence comprehension in DAT patients and found that when working memory demand was kept at a low level, the comprehension of semantically reversible sentences was impaired, whereas the comprehension of nonreversible sentences was not. The findings showed that there was a genuine syntax-specific impairment in DAT which is independent from semantic deficits or working memory dysfunction. According to the Processing Resource Deficit Hypothesis, it is working memory decline that causes the difficulty in sentence comprehension, rather than syntactic decline (Rochon, Waters & Caplan, 1994, 2000; Waters, Caplan & Rochon, 1995). These studies demonstrated that there is little performance decline in DAT patients with the increase of syntactic complexity. In other words, the performance of patients did not differ significantly between syntactically simple sentences and complex ones (Waters, Caplan & Hildebrandt, 1991; Waters & Caplan, 1997). Rochon, Waters & Caplan (1994) maintained that the comprehension deficits observed in the procedures such as the token test or sentence-picture matching merely reflected patients’ performance in the “post-interpretive” process, a process which occurs only when syntactic processing is finished. As the post-interpretive process does not involve syntactic processing, the comprehension decline cannot be attributed to syntactic deficits. Rather, Rochon, Waters & Caplan (1994) found that patients had greater difficulty in comprehending the sentences with more propositions and thus attributed sentence comprehension deficits to the increased semantic and conceptual complexity. Both the Syntactic Deficit Hypothesis and the Processing Resource Deficit Hypothesis have been supported by empirical studies and it is not clear whether sentence comprehension deficit in DAT is the result of syntactic impairment or working memory dysfunction.

Although many studies have investigated sentence comprehension deficits in English-speaking DAT patients, to our knowledge, how sentence comprehension deficits in Chinese-speaking patients manifest has rarely been explored. Chinese is a typologically unique language which is different from Indo-European languages such as English in many dimensions. Although Chinese has the preferred or basic word order of subject–verb–object (SVO), sentences can be arranged in various ways (Xu, 2004). Therefore, the word order of Chinese sentences is relatively more flexible than that of English sentences. Besides, unlike Indo-European languages which have highly elaborate inflectional systems, Chinese has few overt inflectional markers (Li, Bates & Macwhinney, 1993). Another prominent feature of the Chinese language is that it is a tonal language in which tonal variations are used to signal the semantic information of words. As the basis of lexical perception, tonality plays an essential role in the interpretation of spoken sentences in Chinese. However, as the present study focused on the comprehension of written sentences, tonality seems to be less relevant and thus was not be taken into consideration.

The Chinese language, with its flexible word order and lack of inflection, offers the opportunities which are not given in other languages like English, to test the two competing accounts for sentence comprehension deficits in DAT. The importance of studying Chinese-speaking patients became obvious from Zhang, Yu & Boland’s (2010) study which has found that semantic analysis plays a more important role in the processing of Chinese sentences. As semantic processing is typically impaired among DAT patients, Chinese patients might have more severe impairments than English patients. Although this study was not intended to compare the performance of Chinese- and English-speaking DAT patients, the differences outlined above indicate that the findings derived from studies of English-speaking DAT patients are not necessarily applicable to Chinese-speaking patients. Moreover, there are unique sentence constructions in Chinese which have no equivalence in English such as the head-final RCs or *ba* sentences, so the tudies of sentence comprehension in the Chinese language can provide cross-linguistic evidence for sentence comprehension deficits in DAT patients. Therefore, the aim of our study was to investigate sentence comprehension deficits in Chinese-speaking patients with DAT. Specifically, the following two research questions will be addressed:

1. Do Chinese-speaking patients with DAT comprehend sentences significantly differently from the healthy controls?
2. Which of the two hypotheses (Syntactic Deficit Hypothesis or Processing Resource Deficit Hypothesis) can better account for sentence comprehension deficits in Chinese-speaking patients?

## Experiment 1

If we want to find the underlying reasons for sentence comprehension impairments in Chinese patients with DAT, the first step is to identify the specific patterns of comprehension impairments. The first experiment was designed for this purpose. Specifically, there were two objectives of this experiment. First, it aimed to explore how sentence comprehension was impaired in patients with different degrees of dementia severity. Second, it aimed to examine the role of syntactic complexity in sentence comprehension among Chinese-speaking patients with DAT.

For this purpose, we implemented a sentence-picture matching task with two types of Chinese relative clauses (RCs) with different levels of syntactic complexity. The following two research hypotheses were predicted based on the findings from prior research: (1) comprehension depends on the degree of severity. Previous studies have discovered a close association between sentence comprehension performance and the severity of dementia (Grober & Bang, 1995). Therefore, the healthy controls were hypothesized to comprehend sentences most accurately, and the patients with mild DAT were hypothesized to perform more accurately than those with moderate DAT; (2) comprehension is a function of syntactic complexity. As syntactic complexity increases, sentence comprehension becomes more difficult in that it involves more complex syntactic operations and greater demand for working memory resources. Numerous studies have found that syntactically more complex sentences such as ORCs or passives are more difficult to comprehend than simple sentences in DAT patients (Bickel et al., 2000), as well as aphasic patients (Law & Leung, 1998). Therefore, the syntactically more complex structures were hypothesized to be comprehended less accurately than the less complex ones.

## Materials and Methods

### Participants

Forty-four participants were recruited in this experiment, including 22 patients diagnosed with DAT (13 female, age range: 65–84), and 22 age-matched healthy controls (14 female, age range: 61–78). The sample size was determined on the basis of prior research investigating sentence comprehension deficits in patients with DAT. All patients were referred by neurologists from Xuanwu Hospital, Capital Medical University in Beijing and healthy controls were recruited from the local community. All participants were native speakers of Chinese. DAT patients were thoroughly evaluated to determine their dementia status and degree of severity. The evaluation included complete physical and neurological examinations, medical history, neuropsychological assessments and standard laboratory tests. The patients were diagnosed with DAT according to the National Institute of Neurological Disorders and Strokes-Alzheimer’s Disease and Related Disorders Association Task Force criteria (McKhann et al., 1984). The data were reviewed by neurologists who classified the patients as demented or not in accordance with the Diagnostic and Statistical Manual of Mental Disorders 4th Edition criteria for dementia (American Psychiatric Association, 1994). The degree of severity ranged from mild to moderate as measured with the Clinical Dementia Rating scale (CDR; Morris, 1993), which is an instrument used internationally to assess the degree of dementia severity (Perneczky et al., 2006). The CDR involves six cognitive or behavioral domains including memory, orientation, judgment and problem solving, community affairs, personal care, home and hobbies performance. Impairments in these domains (except for the personal care domain) are rated on a five-point scale. The domain scores are then synthesized to assign an overall category of dementia severity. The final categories of severity are: 0 (no dementia), 0.5 (questionable dementia), 1 (mild dementia), 2 (moderate dementia) and 3 (severe dementia). In this study, healthy controls all scored 0 in CDR (Morris, 1993) and lower than level 2 in the Global Deterioration Scale (Reisberg et al., 1982), which suggests that they were all cognitively healthy and free from dementia. Participants were excluded if they reported any history of cerebrovascular disease, brain tumors, history of psychosis, congenital mental growth retardation, severe psychiatric disorders, traumatic brain injury, alcoholism, drug abuse, thyroid dysfunction, severe anemia, syphilis, or HIV. All participants gave verbal informed consent to this investigation and the study was approved by the Ethics Committee of Beijing Foreign Studies University (190808) and implemented according to the Declaration of Helsinki. For all experiments, we have reported all measures, all conditions, all data exclusions, and how sample sizes were determined in this paper. The patients did not differ significantly from the healthy controls in age, *t* (42) = 0.91, *p* = 0.370, education, *t* (42) = 1.17, *p* = 0.248, and gender ratio, χ^2^ = 0. 91, df = 1, *p* = 0.340. Table 1 summarizes the demographic information of the participants.

**Table 1:** Demographic characteristics of AD patients and healthy controls.

	Healthy control (*N*= 22)	DAT (*N*= 22)	DAT subgroups	
			Mild (*N*= 12)	Moderate (*N*= 10)
Age (years)	66.13 (3.75)	67.81 (7.86)	66.16 (7.82)	69.8 (7.84)
Gender (Female/Male)	14/8	13/9	7/5	6/4
Education (years)	12.04 (2.64)	12.9 (2.30)	13 (2.25)	12.8 (2.29)
Duration of DAT (months)	–	24.68 (7.27)	20.83 (4.32)	29.3 (7.57)

**Note:**

C-MMSE, Chinese Mini Mental State Examination.

### Experimental stimuli

RCs were used as experimental sentences in this study. RCs were chosen as experimental stimuli because Kempler et al. (1998) found that RCs were more sensitive to errors in DAT patients compared with other structures such as simple sentences or conjoined sentences. Moreover, as Chinese RCs are uniquely head-final, the study of this structure can provide cross-linguistic evidence for comprehension deficits in DAT. Two types of RCs were created to represent different levels of syntactic complexity, namely the syntactically more complex subject relative clauses (SRCs) and less complex object relative clauses (ORCs). RCs were categorized into high and low complexity according to Gibson’s (1998) Dependency Locality Theory which defines syntactic complexity in terms of the linear distance between the gap and the filler. Table 2 presents the sample experimental sentences and the verbatim English translation.

**Table 2:** Sample sentences used in Experiment 1.

Sentence type	Experimental sentence
SRC (more complex)	Zhuigan nanhai de mama nazhe yusan Chase boy de mother hold-asp umbrella The mother who chases the boy is holding an umbrella
ORC (less complex)	Nanhai zhuigan de mama nazhe yusan Boy chase de mother hold-asp umbrella The mother whom the boy chases is holding an umbrella

**Note:**

SRC, subject relative clause; ORC, object relative clause; (a) experimental sentence; (b) transliteration; (c) English equivalence.

As shown in Table 2, there is a three-word interval between the sentence-initial gap and the filler *mama* in the SRC and there is only one-word interval between the gap (object position of *zhuigan*) and the filler *mama* in the ORC. Therefore, SRCs are considered as syntactically more complex structures and ORCs as less complex structures in our study. As the focus of this study was on syntactic comprehension, syntactic analysis was enforced through the use of semantically reversible sentences because semantic information is not very helpful for the assignment of semantic roles in such sentences and participants need to rely mostly on syntactic information to understand the sentences (Grober & Bang, 1995).

In this design, when two types of RCs had the same head noun *mama* “mother”, they also had the same nouns within the relative clause regions. Both conditions had the same main clause verb (*nazhe* “hold”), main clause object (*yusan* “umbrella”) and RC-internal verb (*zhuigan* “chase”). This design allowed for the minimization of lexical differences across the four conditions. As the two types of RCs contained the same words, they were of the same length. Therefore, sentence length was well matched between the two types of RCs. Together with the oral presentation of every sentence, four pictures were shown to participants, one of which corresponded correctly to the given sentence. The other three pictures were distracters which corresponded to the syntactic foils of the sentence. The sentence “*zhuigan nanhai de mama nazhe yusan*” (The mother who chases the boy is holding an umbrella), for example, was presented with a picture showing the mother who holds an umbrella and chases a boy, a second picture showing a mother chasing a boy who holds an umbrella, a third picture showing a boy chasing the mother who holds an umbrella, and a fourth picture showing a boy who holds an umbrella and chases the mother. A total of 30 sentences were tested, including 15 SRCs and 15 ORCs. Thirty fillers with various syntactic structures were also included. The sentences were presented in a randomized order.

To ensure that there was no difference in the semantic plausibility of the sentences between the two conditions, we designed a sentence plausibility rating survey and invited 28 adults (14 old, 14 young) to rate the plausibility of the experimental sentences using a five-point scale with scores ranging from “1” (the least plausible) to “5” (the most plausible). Those who participated in the survey did not take part in the experiment. A *t*-test was performed to assess the significance of the difference in plausibility ratings between SRCs and ORCs and the results indicated that there was no significant difference between the two types of RCs, SRC: *M* = 4.60; ORC: *M* = 4.66; *t* (27) = −1.24, *p* = 0.225.

### Working memory task

In this study, Daneman & Carpenter’s (1980) experimental paradigm was used to measure subjects’ verbal working memory span. Subjects were instructed to read Chinese sentences which are composed of 16–18 words and recall the final words in the sentences. Then they were required to answer questions which tested their comprehension of the sentences they have read. The sentences we used were from Liu, Wang & Wang’s (2019) study. Subjects were informed that their performance on word recall was as important as that on sentence comprehension. The sentences were presented to subjects in groups, with the number of sentences in each group ranging from two to seven. Subjects were first presented with the two-sentence group, followed by the three-sentence group, four-sentence group and five-sentence group. Subjects completed two practice questions before they proceeded to the formal test. The score was the maximum number of sentences in the group that they both correctly recalled the last words and answered the comprehension questions. The results indicated that there was a significant difference between the healthy controls and the patients in verbal working memory span, *t* (42) = −5.28, *p* < 0.05, with the healthy controls scoring significantly higher than DAT patients.

### Procedure

At the beginning of the experiment, a short pretest was implemented to ensure participants could correctly identify the key words (verbs and nouns) used in the subsequent task. Participants were instructed to identify the picture which depicts the action or person named by the experimenter. If they made a mistake, the experimenter corrected them and retested the word. This procedure was repeated until they could correctly identify all words and then they were allowed to proceed to the formal experiment. In the experiment, they were instructed to listen to a sentence and identify the picture which can best match the meaning of the sentence. They indicated their choice verbally or using hand gesture and the experimenter recorded their answer on an answer sheet. They were required to complete five practice questions before proceeding to the experiment. The experimenter offered assists if they encountered difficulty during their practice. Participants were allowed to listen to the sentence repeatedly and if they still failed to respond, the current trial was rated as incorrect and the next trial began. The experiment took over 20 min and during the experiment, the patients were accompanied by their family who provided them support.

## Results

Table 3 summarized the performance of the patients and healthy controls in Experiment 1. Repeated measures ANOVA was performed with group (healthy control, patient) and syntactic complexity (SRC, ORC) as the predictors and the accuracy of comprehension (percentage of correct answers) as the dependent variable. The results revealed a significant main effect of group, *F*_1_ (1, 42) = 61.06, *p* < 0.05, *F*_2_ (1, 28) = 75.49, *p* < 0.05, a significant effect of syntactic complexity, *F*_1_ (1, 42) = 19.44, *p* < 0.05, *F*_2_ (1, 28) = 20.22, *p* < 0.05 and an interaction between group and syntactic complexity, *F*_1_ (1, 42) = 6.57, *p* < 0.05, *F*_2_ (1, 28) = 6.53, *p* < 0.05. The findings indicate that the healthy controls comprehended sentences more accurately than DAT patients. Pairwise comparison shows that SRCs were comprehended less accurately than ORCs in both DAT patients and healthy controls (*p*s < 0.05). However, a stronger effect of syntactic complexity was found in patients than in healthy controls.

**Table 3:** Descriptive statistics for comprehension accuracy (Experiment 1).

Type	Group						*F*	Sig. (*p*)	η_*p*_^2^
	Control		Mild DAT		Moderate DAT				
	*M*	SD	*M*	SD	*M*	SD			
SRC	96.1%	0.04	84.4%	0.11	73.3%	0.05	35.84	<0.001	0.636
ORC	98.7%	0.03	91.7%	0.08	87.3%	0.09	12.81	<0.001	0.385

**Note:**

SRC, subject relative clause; ORC, object relative clause.

**Figure 1:**
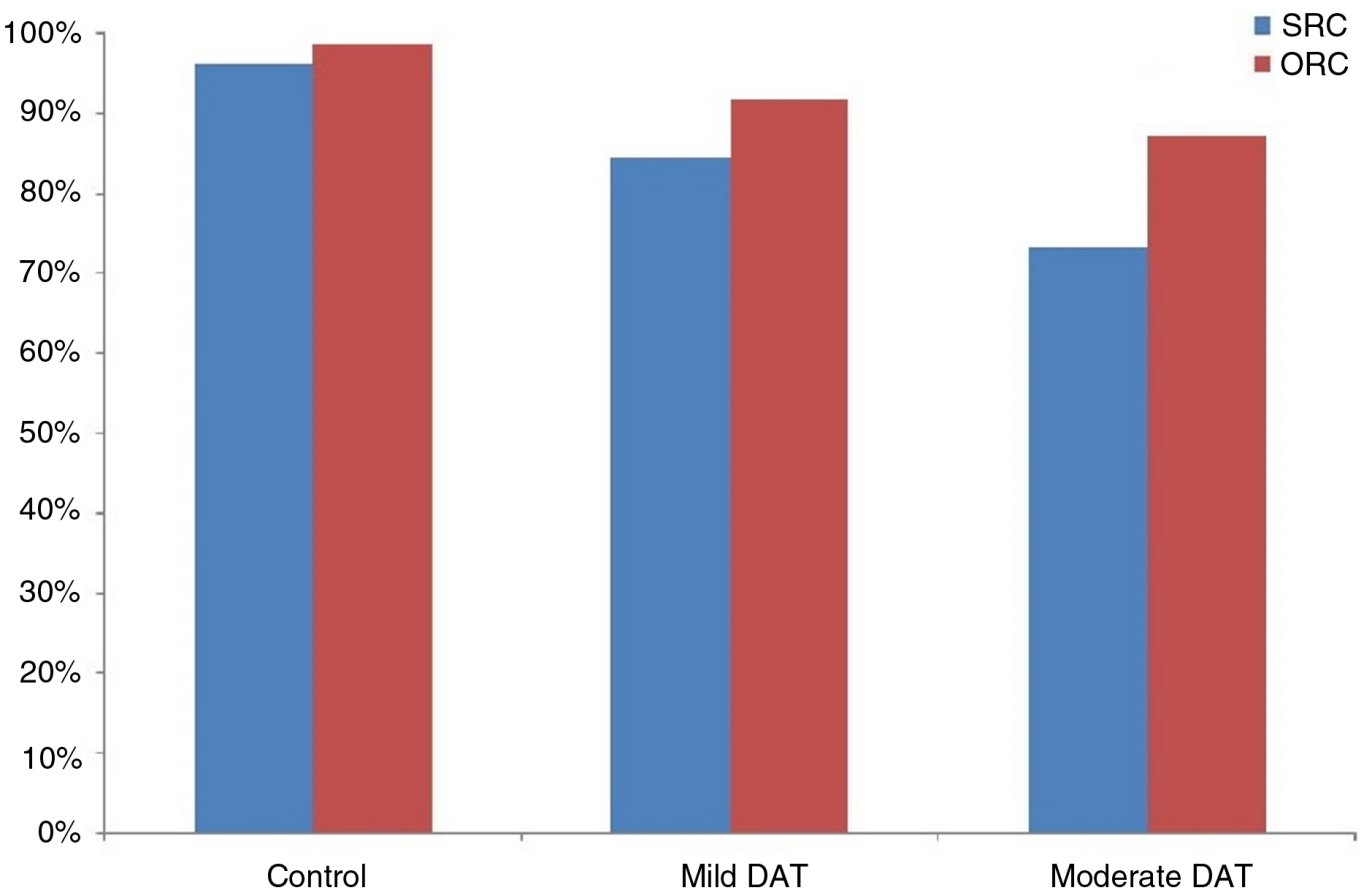
Accuracy of sentence comprehension by patients and healthy controls (Experiment 1).

The performance of patients with different severity is graphically summarized in Fig. 1. To explore the relationship between degree of severity and syntactic complexity, we performed a repeated measures ANOVA with severity (mild, moderate) and syntactic complexity (SRC, ORC) as the predictors and accuracy of comprehension (percentage of correct answers) as the dependent variable. The results revealed a significant effect of severity, *F*_1_ (1, 20) = 8.90, *p* < 0.05, *F*_2_ (1, 28) = −8.17, *p* < 0.05, a significant effect of syntactic complexity, *F*_1_ (1, 20) = 14.21, *p* < 0.05, *F*_2_ (1, 28) = −14.39, *p* < 0.05. The interaction between group and syntactic complexity was not significant, *F*_1_ (1, 20) = 1.45, *p* = 0.243, *F*_2_ (1, 28) = −1.57, *p* = 0.220. The patients with moderate DAT performed worse than the mild group, which suggests that sentence comprehension abilities decline with the progress of the disease. SRCs were comprehended less accurately than ORCs in both groups of patients.

To explore whether the observed effect of syntactic complexity was related to working memory span, we adopted Caplan & Waters’s (2005) approach to examining the correlation between working memory span and the accuracy difference scores between syntactically more complex sentences and less complex sentences. The difference in accuracy between SRCs and ORCs was calculated and used as an index of the increased difficulty in comprehending the syntactically more complex SRCs. Negative difference scores in accuracy indicates an effect of syntactic complexity (Caplan & Waters, 2005). The results of Spearman correlation analysis revealed a significant negative relationship between the accuracy difference scores and working memory span for the healthy controls, *r*_s_ = −0.54, *p* < 0.05, the patients with mild DAT, *r*_s_ = −0.63, *p* < 0.05, as well as the patients with moderate DAT, *r*_s_ = −0.81, *p* < 0.05, which suggests that in all three groups, subjects with smaller working memory span were more affected by the structural difference between SRCs and ORCs.

## Discussion

Results of Experiment 1 show that compared with healthy controls, DAT patients comprehended sentences less accurately. Compared with healthy older adults, syntactic complexity had a stronger effect on DAT patients. The findings of Experiment 1 are largely consistent with previous studies which also found significant deficits in sentence comprehension in DAT patients speaking other languages such as English (Bates, Wulfeck & MacWhinney, 1991; Grober & Bang, 1995; Kempler et al., 1998; Emery, 1985; Grober & Bang, 1995; Kontiola et al., 1990; Leikin & Aharon Peretz, 1998; MacDonald et al., 1996; Rochon, Waters & Caplan, 1994; Small, Kemper & Lyons, 2000; Tomoeda et al., 1990; Marková et al., 2017).

The study also revealed that the impairment in sentence comprehension became increasingly severe as the severity of the illness increased from mild to moderate. Concerning the effect sizes for the difference in accuracy scores between the three groups of participants, the observed η_*p*_^2^ values shown in Table 3 fall into the large range for both SRCs and ORCs (small effect: 0.01, medium effect: 0.06, large effect: 0.14, Cohen, 1988). The effect size analyses demonstrated that there was a significant difference between the three groups in the accuracy of sentence comprehension. Moreover, the effect of syntactic complexity was magnified with the increase of severity. The patients with more severe DAT had greater difficulty in comprehending syntactically complex sentences, a finding in line with Grober & Bang’s (1995) study. As the sentence-picture matching task was an offline task, participants needed to memorize the meaning of the sentences in order to correctly match them with the pictures. Therefore, this type of tasks induce high working memory burden, which might explain why the patients performed significantly worse than the healthy controls. This has been confirmed by the significant correlation between the accuracy difference scores and working memory scores. In healthy controls, mild and moderate patients, working memory has been found to correlate negatively with the accuracy difference scores which suggests that working memory is a universal constraint on sentence comprehension for both healthy older adults and DAT patients. This finding was supported by Alatorre-Cruz et al.’s (2018) study which have found that older adults showed problems in sentence processing when working memory load was increased. According to Evans’s (1996) suggested guide for the absolute value of correlation coefficient (weak: *r* = 0.20–0.39, moderate: *r* = 0.40–0.59, strong: *r* = 0.60–0.79, very strong: *r* = 0.80–1.0), the correlation between the accuracy difference scores and verbal working memory span was moderate in the healthy controls, strong in the patients with mild DAT and very strong in the patients with moderate DAT. These findings indicate that there was a close association between the observed syntactic complexity effect and verbal working memory span in both healthy controls and DAT patients. The connection between syntactic complexity effect and working memory span seemed to get stronger with the progression of the disease from mild to moderate severity, which suggests that working memory dysfunction might be an increasingly important source of sentence comprehension deficits with the increase of dementia severity.

In this study, DAT patients comprehended SRCs with greater difficulty than ORCs, a finding which is contrary to studies from English-speaking patients which have found that ORCs were more difficult to comprehend than SRCs (Bickel et al., 2000; Kempler et al., 1998). This discrepancy mainly results from the structural difference between English and Chinese RCs. As Chinese RCs are head-final structures with relative clauses coming before the head nouns, the linear distance between the gap and the filler is greater in SRCs than that in ORCs. According to Gibson’s (1998) Dependency Locality Theory, SRCs are syntactically more complex than ORCs in Chinese. This finding agrees with the studies of RC comprehension in Chinese-speaking aphasic patients which have also demonstrated that SRCs were more difficult to comprehend than ORCs (Law & Leung, 1998; Su, Lee & Chung, 2007; Zhou et al., 2010).

Findings from Experiment 1 provided evidence showing that Chinese-speaking patients had great difficulty in the comprehension of RCs. As this study made the first attempt to explore sentence comprehension deficits among Chinese patients with DAT, it offered important information which can further our understanding of language deficits related to DAT. However, although the findings of this experiment make it possible for us to infer the crucial association between working memory and sentence comprehension deficits in DAT patients, we cannot exclude the possibility that the decline in syntactic ability might also contribute in some way to their performance in sentence comprehension. Therefore, we implemented a second experiment to explore the reasons for sentence comprehension deficits in Chinese-speaking patients with DAT.

## Experiment 2

The aim of Experiment 2 was to investigate whether working memory decline and syntactic decline contributed to sentence comprehension deficits in DAT patients. We modified the procedures of Experiment 1 to reduce the working memory burden in sentence comprehension. This experiment differed from Experiment 1 in that the sentences and pictures were presented visually at the same time and subjects were allowed to review them at their own pace. In this way, the working memory burden of the task was considerably reduced because subjects did not have to memorize the meaning of the sentences during their selection of the pictures. The basic assumption is that if the effect of syntactic complexity is significant after working memory burden is minimized, the impairment should be attributed to syntactic deficits. Contrarily, if the effect of syntactic complexity becomes insignificant after working memory burden is minimized, the impairment should be attributed to working memory dysfunction, rather than syntactic decline.

## Method

### Participants

A total of 18 participants diagnosed with DAT (10 mild and eight moderate, 10 female, age range: 59–84) and 20 age-matched healthy controls (14 female, age range: 61–78) participated in this experiment. The sample is a subgroup of the participants in the first experiment (refer to Table 1 for demographic details and see above for diagnostic details). Participants were excluded if they reported any history of reading difficulty. No significant difference was found between the healthy controls and the patients in age, *t* (36) = 1.06, *p* = 0.296, years of education, *t* (36) = 1.31, *p* = 0.197, or gender ratio, χ^2^ = 0.85, df = 1, *p* = 0.357. The healthy controls were found to have larger working memory span than the patients with DAT, *t* (36) = −5.32, *p* < 0.05.

### Experimental stimuli

The sentences used in Experiment 2 are also Chinese ORCs and SRCs. However, a different set of sentences were used. The sentences included 15 SRCs and 15 ORCs, along with 30 fillers of various structures and length. Table 4 provides an example of the sentences used in this experiment. The stimuli were developed by reordering the linguistic components of the sentences used in Experiment 1. For example, the sentences in Table 3 were created by reversing the order of RC subjects and RC objects in the sentences in Table 2. The sentences used in the two experiments shared the same lexical components, but they differed in meanings.

**Table 4:** Sample sentences used in Experiment 2.

Sentence type	Experimental sentence
SRC (more complex)	Zhuigan mama de boy nazhe yusan Chase mother de boy hold-asp umbrella The boy who chases the mother is holding an umbrella
ORC (less complex)	Mama zhuigan de nanhai nazhe yusan Mother chase de boy hold-asp umbrella The boy whom the mother chases is holding an umbrella

**Note:**

SRC, subject relative clause; ORC, object relative clause; (a) experimental sentence; (b) transliteration; (c) English equivalence.

### Procedure

The subjects were tested on an individual basis. After the pretest on the meaning of nouns and verbs in the experimental sentences, subjects were instructed to look at a sentence and identify the picture which can best match the meaning of the sentence. The subjects indicated their choices using hand gesture or verbally and the experimenter recorded their answers on an answer sheet. Subjects could refer to the written sentence for as many times as possible and if they failed to respond to the question, the next trial began. The subjects completed five practice questions before proceeding to the formal experiment. The experiment lasted for over 20 min, during which patients were accompanied by their family who gave them support.

## Results

Table 5 summarized the performance of the patients and healthy controls in Experiment 2. ANOVA was applied with the proportion of correct answers as the dependent variable and group (healthy control, DAT patient) and syntactic complexity (SRC, ORC) as independent variables. The results revealed a significant effect of group, *F*_1_ (1, 36) = 59.80, *p* < 0.05, *F*_2_ (1, 28) = 190.80, *p* < 0.05, a significant effect of syntactic complexity, *F*_1_ (1, 36) = 8.01, *p* < 0.05, *F*_2_ (1, 28) = 6.33, *p* < 0.05 and a significant interaction between group and syntactic complexity, *F*_1_ (1, 36) = 12.84, *p* < 0.05, *F*_2_ (1, 28) = 19.16, *p* < 0.05. The healthy controls comprehended sentences more accurately than patients. Pairwise comparison showed that there was a significant effect of syntactic complexity in patients with DAT, with SRCs comprehended significantly less accurately than ORCs. However, the effect of syntactic complexity was not significant in the healthy controls (*p* = 0.453).

**Table 5:** Descriptive statistics for comprehension accuracy (Experiment 2).

Type	Group						*F*	Sig. (*p*)	η_*p*_^2^
	Control		Mild DAT		Moderate DAT				
	*M*	SD	*M*	SD	*M*	SD			
SRC	98.6%	0.03	88.1%	0.06	67.5%	0.07	113.31	<0.001	0.866
ORC	97.6%	0.04	90.7%	0.06	83.4%	0.06	22.85	<0.001	0.566

**Note:**

SRC, subject relative clause; ORC, object relative clause.

**Figure 2:**
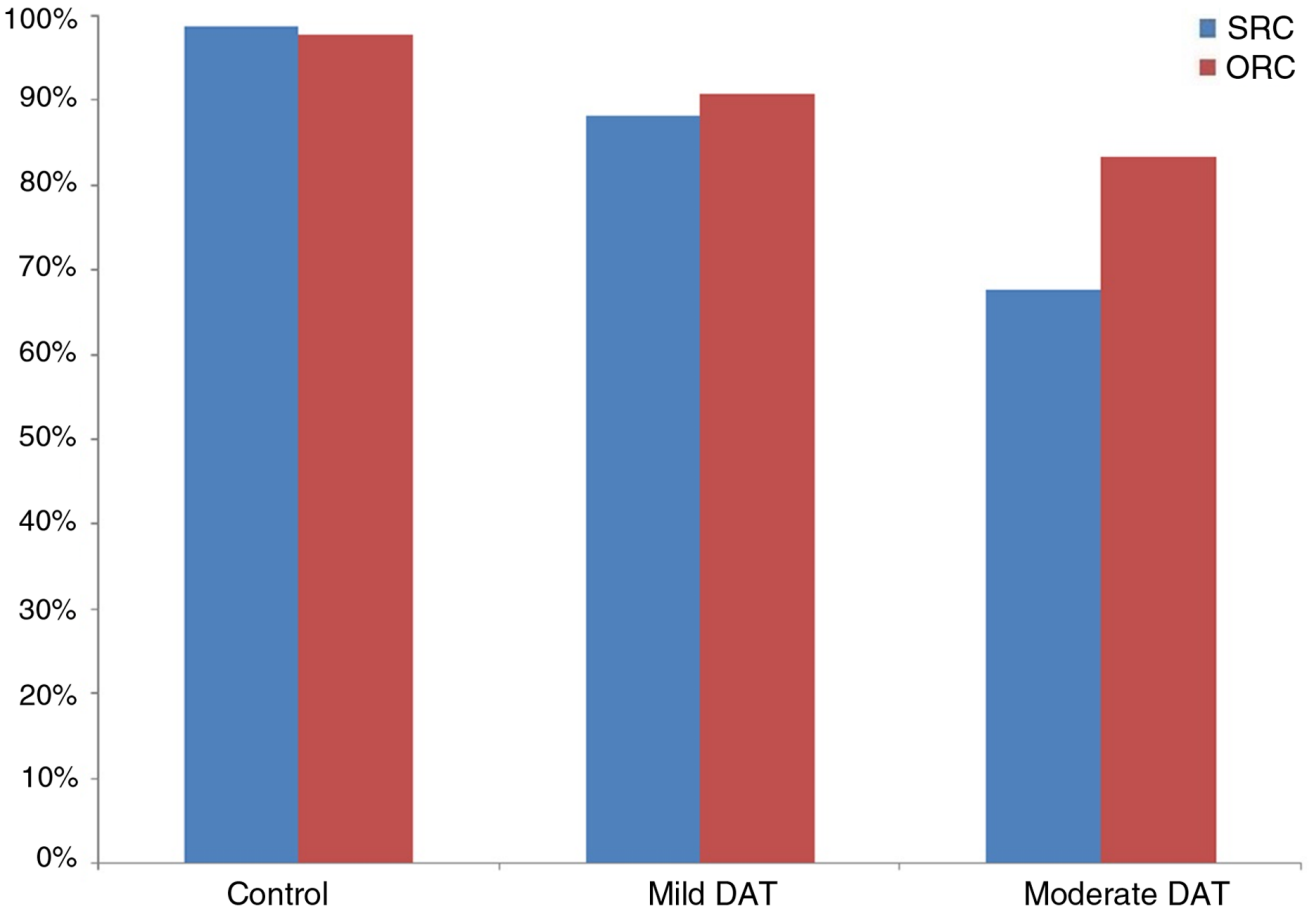
Accuracy of sentence comprehension by patients and healthy controls (Experiment 2).

The performance of patients with different degrees of dementia severity is summarized in Fig. 2. To explore the relationship between degree of severity and syntactic complexity, an ANOVA was performed with severity (mild, moderate) and syntactic complexity (SRC, ORC) as the predictors and percentage of correct answers as the dependent variable. The results revealed a significant effect of severity, *F*_1_ (1, 16) = 34.94, *p* < 0.05, *F*_2_ (1, 28) = 18.91, *p* < 0.05, a significant effect of syntactic complexity, *F*_1_ (1, 16) = 24.58, *p* < 0.05, *F*_2_ (1, 28) = 29.40, *p* < 0.05 and a significant interaction between severity and syntactic complexity, *F*_1_ (1, 16) = 12.45, *p* < 0.05, *F*_2_ (1, 28) = 6.58, *p* < 0.05. The patients with moderate DAT comprehended the sentences significantly less accurately than those with mild DAT. Pairwise comparison suggested that no significant difference between SRCs and ORCs was found in the patients with mild DAT (*p* = 0.223), although patients in the moderate group comprehended ORCs significantly more accurately than SRCs.

A Spearman correlation analysis was performed to understand the observed effect of syntactic complexity in the patients with moderate level of severity. The results showed that in patients with moderate DAT, the difference in accuracy between SRCs and ORCs was not correlated significantly with verbal working memory span, *r*_s_ = 0.66, *p* = 0.071, which suggested that the effect of syntactic complexity observed in the patients with moderate DAT was not closely associated with working memory decline.

## Discussion

Experiment 2 aimed to explore the reasons for sentence comprehension deficits in DAT patients. The finding from this experiment showed that when working memory burden was minimized in the sentence-picture matching task, the healthy controls and patients with mild DAT were no longer disrupted by syntactic complexity. For these two groups of participants, there was no significant difference between SRCs and ORCs in the accuracy of comprehension. The moderate group, however, were still affected by syntactic complexity. Results of correlation analysis indicate that the effect of syntactic complexity in patients with moderate DAT could not be attributed to working memory decline as there was no significant correlation between working memory span and the accuracy difference scores. This finding suggests that there was a syntactic-specific decline in the moderate group, which was independent from working memory dysfunction. The findings from this experiment show that working memory decline is the source of comprehension deficits for patients with mild DAT and syntactic decline is one of the reasons for the deficits manifested among patients with moderate DAT. If the findings from Experiment 1 are taken into consideration, we may find that the source of impairments in patients with moderate DAT is not just syntactic decline, but also working memory decline. Given the findings from Experiment 1 that working memory was correlated with the effect of syntactic complexity in both groups of DAT patients, working memory seems to be a universal constraint on the performance of all patients, regardless of their degree of severity. In this way, although patients with mild DAT were affected by working memory decline alone, those with moderate DAT were influenced by both working memory dysfunction and syntactic decline. This study provided a fine-grained picture about the relationship between the source of comprehension deficits and degree of severity.

With the progression of the disease, patients became increasingly vulnerable in syntactic comprehension. These findings are in agreement with those of Bickel et al.’s (2000) study which has examined syntactic comprehension in German-speaking patients and discovered that syntactic ability was mildly affected at the early stages of Alzheimer’s disease and was rather severely impaired at the more advanced stages. However, the findings are inconsistent with Grossman et al. (1995) and Waters, Caplan & Rochon’s (1995) research which did not find significant relationships between sentence comprehension deficits and degree of severity as measured by Mini-Mental State Examination (MMSE). The divergence might result from the different ways of categorization in terms of degree of dementia severity. Our study used the CDR to divide patients into mild and moderate groups, whereas Grossman et al. (1995) and Waters, Caplan & Rochon’s (1995) classification was based on the MMSE (Folstein, Folstein & McHugh, 1975). Taken together, these studies indicate that sentence comprehension deficits might be independent from the general cognitive processes measured by MMSE (Croot, Hodges & Patterson, 1999), but more related to the cognitive and functional performance assessed by CDR.

The findings provided mixed support for the Syntactic Deficit Hypothesis and the Processing Resource Deficit Hypothesis. Specifically, the findings related to mild DAT support the Processing Resource Deficit Hypothesis, but those relevant to the moderate DAT seem to support the Syntactic Deficit Hypothesis as well as the Processing Resource Deficit Hypothesis. This is consistent with many previous studies which have found working memory to be a major reason for sentence comprehension deficits in DAT patients (Rochon, Waters & Caplan, 1994; Small, Andersen & Kempler, 1997; Waters, Caplan & Hildebrandt, 1991; Waters, Caplan & Rochon, 1995), and the typical observation that syntactic abilities are only impaired at the later stage of DAT (Bayles, 1982; Bayles & Boone, 1982; Hier, Hagenlocker & Shindler, 1985; Kertesz, Appell & Fisman, 1986; Miller, 1989). These results suggest that the sentence comprehension deficits in patients with different degrees of severity were attributed to different factors. Syntactic ability was relatively intact in patients with mild DAT and their sentence comprehension deficits mainly resulted from the insufficiency in working memory resources. In the moderate group, however, comprehension deficits were caused by both working memory dysfunction and syntactic decline. The findings might be related to the progressive brain atrophy which characterized the development of DAT. Brain imaging studies discovered that cerebral atrophy is well established by the time patients are diagnosed as DAT (Johnson et al., 2012; Leung et al., 2013). Even for patients with mild DAT, hippocampal volumes have dropped by 15–25% and entorhinal volumes by 20–30% (Chan et al., 2001; Dickerson et al., 2001; Schuff et al., 2009). For patients with moderate or severe DAT, hyppocampal volumes and entorhinal volumes drop to an even greater extent. As both entorhinal cortex and hippocampal cortex play an essential role in memory, the atrophy in these two regions inevitably lead to working memory deficits in all DAT patients regardless of their degree of severity. As the disease progresses to moderate severity, the areas of volume loss become more extensive, with multiple areas showing increased rate of atrophy including the temporal lobes, precuneus, the posterior cingulate gyrus and anterior cerebral cortices (Scahill et al., 2002). Previous neuroimaging studies have identified many of these areas as the sites for syntactic capacity such as anterior, middle and superior temporal lobe structures (Kaan & Swaab, 2002), cingulate gyrus (Ansaldo et al., 2015; Ashtari et al., 2005) and anterior cerebral cortices. The cerebral atrophy in these areas may lead to greater difficulties in syntactic comprehension for patients with moderate DAT. The progression of DAT can lead to the impairment of an increasing number of cerebral cortex, which can cause decrements of both general cognitive ability and linguistic abilities. This might explain why patients with moderate DAT have showed multiple sources for sentence comprehension deficits. However, so far neuroimaging studies of DAT have not given particular attention to the brain atrophy related to specific language impairments. Further research is needed to help us understand the relationship between cerebral atrophy and sentence comprehension deficits in DAT.

Findings from the present study have crucial implications for the diagnosis and treatment of DAT. Since this study revealed that there is syntactic impairment merely in patients with moderate DAT, the presence of such impairment might be considered as a sign showing that the patients have progressed into a moderately severe stage. Therefore, syntactic decline might be used as a criterion for categorizing DAT patients into different degrees of severity. Besides, the differential impairment of syntactic ability and working memory might be helpful for us to identify the priority of clinical intervention of language disorder in DAT. For example, for patients at the early stage of DAT, the focus of intervention should be placed on the techniques which compensate for their working memory impairments (Small, Andersen & Kempler, 1997; Kumar et al., 2017), rather than on linguistic impairments. For patients with moderately severe DAT, however, it might not be sufficient to solely implement working memory-based intervention as the present study found that both syntactic decline and working memory dysfunction compromise the patients’ performance in sentence comprehension. Both language- and memory-based interventions are necessary for the patients with moderately severe DAT.

This study is not without limitation. As the present study has only examined the comprehension of SRCs and ORCs, further investigation is needed to explore the performance of DAT patients in comprehending other types of RCs such as *suo* relative clauses and other syntactic structures such as passives. A comprehensive evaluation of various syntactic structures is necessary before we can find out whether syntactic decline is a contributing factor to sentence comprehension impairments in DAT patients. Besides, as the tasks used in this study are offline in nature, they are not very helpful in identifying the comprehension difficulty in online processes. As different working memory resources may be involved in online and offline sentence processing, DAT patients might manifest a different pattern of impairment in online processing. Therefore, further studies are needed to explore how pathological aging may affect online sentence processing and which factors contribute to this process.

## Conclusions

The present study compared the performance in sentence comprehension between healthy controls and DAT patients with mild and moderate severity using a sentence-picture matching paradigm. The results show that the ability to comprehend sentences is clearly decreased in DAT patients. A subdivision into mildly and moderately impaired DAT patients revealed that mildly impaired patients were less affected by syntactic complexity than moderately impaired patients. Working memory decline is the primary source of sentence comprehension impairments for patients with mild DAT and both syntactic decline and working memory decline contribute to sentence comprehension deficits in patients with moderate DAT. The findings provided mixed support for the Syntactic Deficit Hypothesis and the Processing Resource Deficit Hypothesis.

## Supplemental Information

10.7717/peerj.8181/supp-1 Supplemental Information 1 Performance of AD patients in sentence comprehension.

## Additional Information and Declarations

### Data Availability

The following information was supplied regarding data availability: The measurements are available as a Supplemental File.
